# Correction: The effect of poor vision on economic farm performance: Evidence from rural Cambodia

**DOI:** 10.1371/journal.pone.0315520

**Published:** 2024-12-05

**Authors:** Frederik Sagemüller, Selina Bruns, Oliver Mußhoff

In Fig 1, the textbox under outcome is incorrect. It should have been reduced agricultural productivity. Please see the correct Fig 1 here.

**Fig 1:**
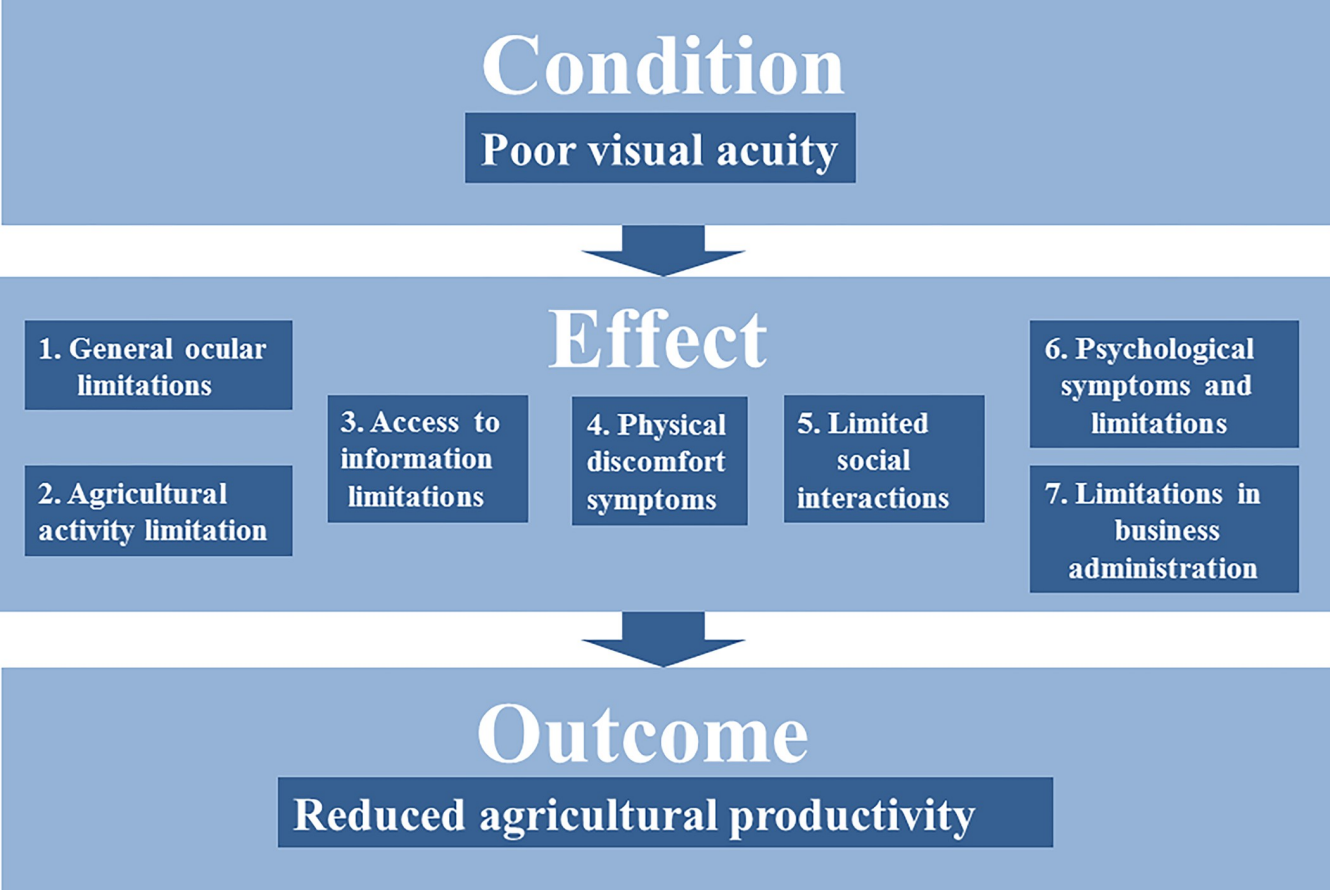
Poor visual acuity and its negative effects on agricultural profitability.

The effects are derived from Kandel et al. (6), themes 1, 3, 4, 5, 6 and 7. Theme 2 was excluded because it deals exclusively with the negative effects of wearing glasses, contact lenses and corrective surgery. Source: Own depiction.
