# Supplementary material for: The effect of poor vision on economic farm performance: Evidence from rural Cambodia
Source: PLoS One. 2022 Sep 9;17(9):e0274048. doi: 10.1371/journal.pone.0274048 (PMC9462746; doi:10.1371/journal.pone.0274048)
Supplement: S1 File — Adapted from Kandel et al. (2018). (DOCX) [file pone.0274048.s001.docx]

**Supporting information S1**

Symptoms and limitations imposed on rural dwellers by poor visual acuity. Adapted from Kandel et al. (2018).

**1. General ocular limitations (41 mentions):** Blurred vision, Cloudy vision, Dazzled vision, Sensation like having a layer or net over the eyes, Distorted vision, Blurred vision at distance, Blurred vision at near, Recognizing objects at far, Reading at far, Seeing things in a relative motion, Headaches, Giddiness or dizziness, Nausea or vomiting, Using my other senses, Floaters in my vision, Flashes of light from within my eyes, Glare from lights, Sensitivity to light, Haloes around lights, Starbursts, Adapting to changes in light, Plain/even roads look like uneven, Prolonged reading, Blurred vision in rain, Poor vision in dim light, Double vision, Seeing objects with ghosts or shadows around them, Difficulty distinguishing colors, Difficulty focusing my eyes, Reading in dim light, Working in dim light, Writing, Riding a motorcycle, Riding, Riding a motorcycle in unfamiliar areas, Riding motorcycle/moped, Walking, Walk, Walking in dawn or dusk, Walking on uneven ground and negotiating bumps or cracks in my path, Going uphill or downhill

**2. Agricultural Activity Limitation (19 mentions):** Seeing small insect pests in vegetables, Seeing insects while doing agricultural works, Finding insect, Bending down, Looking after domestic animals or pets, Cutting grass, Carrying loads, Harvesting grains, Winnowing rice, Weeding, Seeing small insect pests in vegetables, Doing agricultural works like working in the fields, Finding something when it is surrounded by a lot of other things, Using microscope, Reading medicine bottle, Bright sunlight, Cutting or chopping food, Using hand tools like screwdriver and hammer, Avoiding some tasks

**3. Limitations in access to information (18 mentions):**

Reading power point projected slides, Reading small print, Reading things written on a whiteboard, Telling the time from a clock, Reading the newspaper, Watching television, Reading a watch, Reading store names, Reading hoarding boards, Reading cook books, Reading magazines, Reading a book, Reading the phone book, Using a mobile phone, Reading large print, Using the computer, Reading my posts, Reading numbers or letters on the front of a bus or a motorcycle

**4. Physical discomfort symptoms and limitations (18 mentions):**

Squinting or squeezing my eyes, Feeling ill, Feeling like loss of balance, Discomfort in eyes, Dry eyes, Burning in eyes, Watery eyes, Grittiness in eyes, Red eyes, Stinging in eyes, Itchy eyes, Discharge in eyes, Loss of peripheral vision, Swelling of eyelids, Tired eyes, Heavy eyes, Pain in eyes, Poor vision in only one eye

**5. Limited social interactions (18 mentions):**

A crowded environment, Using public transport, Attending social functions, Participating in social activities at night, Meeting friends or family socially, Meeting people for the first time, Getting help and support, Maintaining usual social activities, Maintaining my roles and responsibilities in the family, Meeting someone for the first time, Group activities, People not understanding my eye condition, Taking part in recreational activities, Recognizing someone across the street, Recognizing faces and objects on a photograph, Avoiding class room, conference hall

**6. Limitations in business administration (4 mentions):**

Reloading money on a mobile phone using a recharge card, Difficulty reading a wall-mounted calendar, Signing/putting on a signature, Writing on a cheque

**7. Psychological symptoms and limitations (12 mentions):**

Feel worried, Feel disabled, My eyesight getting worse, Going blind, My prescription (strength of glasses) getting worse, Not knowing what’s going to happen in the future, Fear of falling, Fear of tripping, Fear of getting lost, Feel afraid, Feel nervous, Feel depressed
