# Supplementary material for: The effect of poor vision on economic farm performance: Evidence from rural Cambodia
Source: PLoS One. 2022 Sep 9;17(9):e0274048. doi: 10.1371/journal.pone.0274048 (PMC9462746; doi:10.1371/journal.pone.0274048)
Supplement: S2 File — (DOCX) [file pone.0274048.s002.docx]

Inclusivity in global research

PLOS’ policy on inclusivity in global research aims to improve transparency in the reporting of research performed outside of researchers’ own country or community and ensures that PLOS publications reporting global research adhere to high standards for research ethics and authorship. Authors of relevant research articles may be asked to complete the questionnaire below, which outlines ethical, cultural, and scientific considerations specific to inclusivity in global research. This questionnaire may be requested when researchers have travelled to a different country to conduct research, if research uses samples collected in another country, research with Indigenous populations or their lands, or if research is on cultural artefacts. Researchers travelling to another country solely to use laboratory equipment will not normally be required to complete the questionnaire. However, the questionnaire can be requested at the journal’s discretion for any submission – if you have been requested to complete this questionnaire by the PLOS journal you submitted to, please do so.

Please complete the questionnaire below and include this as a Supporting Information file with your manuscript. Note that if your paper is accepted for publication, this checklist will be published with your article in the supporting information files. Please ensure that you reference the checklist in the main body of your manuscript. We suggest adding a subsection ‘Inclusivity in global research’ to your Methods section and adding the following sentence: “Additional information regarding the ethical, cultural, and scientific considerations specific to inclusivity in global research is included in the Supporting Information (SX Checklist)”

The questions have been designed to be applicable to a wide range of study types, and there are subsections for both human subjects research and non-human subjects research. If any of the questions are not relevant to your research please mark them as “N/A” as appropriate.

**Ethical considerations, permits and authorship**

*This section is applicable to all research types.*

Provide details as to who granted permissions and/or consent for the study to take place in the Methods section of your manuscript. This should include the names of **all** ethics boards, governmental organizations, community leaders or other bodies that provided approval for the study. If individuals provided approval refer to these people by their role or title but do not list their name(s).

Reported on page number: 8

*When we conducted the fieldwork for this project in 2018, the University of Göttingen only had an internal review board system in place for clinical trials. Since our research does not qualify as a clinical trial, we were not eligible for an internal review. Anyhow, in close collaboration with our partners at CIAT (The International Center for Tropical Agriculture) and the Royal University of Agriculture in Phnom Penh, we designed this research project according the principles of ethic responsibility in research involving human subjects and the national legislation of Cambodia. Since 2020, the University of Göttingen has an ethics committee that reviews and approves research that involves human subjects. We submitted our research protocol to them and obtained a retrospective approval. The approval letter was submitted to the editors.*

If there were any deviations from the study protocol after approval was obtained please provide details of these changes in the Methods section of your manuscript.
Did this study involve local collaborators that are residents of the country where the research was conducted or members of the community studied? If you do not have any authors from said communities, please provide an explanation for this below.

Reported on page number:

N/A

*The research was conducted as part of the collaborative research project: “Hands and Minds connected to boost Eco-efficiency on Smallholder Livestock-Crop Systems”, led by the International Center for Tropical Agriculture (CIAT) and in cooperation with - among other local partners - the Royal University of Agriculture (RUA), Phnom Penh, Cambodia and the University of Göttingen, Germany. We engaged in collaborations with local partners as they were part of our CIAT project team. However, the support was exclusively of logistical nature, which does not meet the criteria for authorship. The partners helped us recruit enumerators, with mobility questions, and by introducing us to further local partners such as the commune leaders, local governmental extension offices and village chiefs. Regarding the involvement of local collaborators as authors, we published a collaborative article with researchers from all partner institutions. The present study is a small side project of two German PhD students with their supervisor.*

Everyone listed as an author should meet PLOS’ criteria for authorship and all individuals who meet these criteria should be included in the author byline, rather than the acknowledgements. Authorship criteria is based on the International Committee of Medical Journal Editors (ICMJE) Uniform Requirements for Manuscripts Submitted to Biomedical Journals - for further information please see here: <https://journals.plos.org/plosone/s/authorship>.

**Human subjects research (e.g. health research, medical research, cross-cultural psychology)**

Did you obtain written informed consent from a representative of the local community or region before the research took place? How did you establish who speaks for the community? Details of written informed of consent obtained from study participants should be reported separately in the Methods section of the manuscript.

*First of all, our research was coordinated with the researchers at the CIAT- Hanoi office who managed the larger project on sustainable farming practices in the region. Before visiting the villages, we met the commune leader and the government extension officers of the respective sub-province. The extension officer then accompanied us to each village and introduced us to the village chief, whom we presented our research endeavor in order to get his/her consent for data collection in his/her village. Only after receiving verbal consent from a) the commune chief, b) the extension officer, and c) the village chief were we able to undertake the data collection in the respective village.*

*Before the survey, we explained to each participant what the research is about, what their participation in the project entails and that participation is voluntary. After this was understood we gathered written consent from each participant to be included in the research project. As a compensation for their lost time, the participants where paid equal to half a day of paid labor.*

How did members of the local community provide input on the aims of the research investigation, its methodology, and its anticipated outcome(s)?

*We undertook a qualitative research before starting our quantitative data collection. Through the qualitative insights we developed the quantitative questionnaire. Furthermore, the lead author lived in Cambodia for a considerable amount of time and observed the particularly striking absence of glasses among the rural population.*

When engaging with the local community, how did you ensure that the informed consent documents and other materials could be understood by local stakeholders?

*We employed students from the Royal University of Agriculture who spoke English and Khmer to enable us to communicate with the farmers as well as other local stakeholders. We carried out intensive training sessions on survey methods which included the importance of explaining our research and obtaining informed written consent. One researcher was always present during data collection and she checked every questionnaire and if the protocol for obtaining consent was followed.*

Will the findings of the research be made available in an understandable format to stakeholders in the community where the study was conducted (e.g. via a presentation, summary report, copies of publications, etc.)? Please provide details of how this will be achieved.

*The results from this publication will be shared with the members of the larger CIAT project. Most importantly, we will share the results with the local researchers and project coordinators at the Royal University of Agriculture in Phnom Penh. In collaboration with the local Governmental extension office they frequently conducted field research, farmer trainings and workshops in the local communities to share the results of the research project. Since the research project already ended, we cannot guarantee to carry out similar formats to share our results.*

**Non-human subjects research using specimens/ animals collected as part of the study, or those housed in archival collections. Examples include archaeology, paleontology, botany and zoology.**

Did the permission you obtained from a local authority to perform the study include an agreement on access to outputs and benefit sharing? This may include procedures to enable fair distribution of the benefits and resources arising from the research performed. Please include any details of Prior Informed Consent and Benefit Sharing Agreements obtained. These may be required by field-specific regulations, for example the Convention on Biological Diversity (CBD) and the associated Nagoya Protocol.

N/A

If the material used in your study was imported, please A) provide the year it was imported and B) indicate whether permits were obtained to import/export the materials used, C) provide details of any permits obtained. If this information is not available, please indicate this.

N/A

If you used archival specimens, please state how the material used in your study was acquired by the institute it is held in and provide details of any permits obtained for the original excavations/ sample collection. If this information is not available, please indicate this.

N/A

How was the potential cultural significance of the materials collected in your study to local communities considered in your research design? Were Indigenous peoples and/or local researchers and institutions involved with archaeological excavations / collection of specimens? If so, please provide a description of their involvement.

N/A

If your manuscript includes photographs of human remains please indicate whether authors obtained permission from descendants or affiliated cultural communities to do so.

N/A
