# Supplementary material for: The effect of poor vision on economic farm performance: Evidence from rural Cambodia
Source: PLoS One. 2022 Sep 9;17(9):e0274048. doi: 10.1371/journal.pone.0274048 (PMC9462746; doi:10.1371/journal.pone.0274048)
Supplement: S1 Table — (DOCX) [file pone.0274048.s003.docx]

**Supporting information S2**

S2 Table: Logit model of eyesight with age as only predictor

| Eyesight | Coef. | SE | z | P>z | 95% CI | |
| --- | --- | --- | --- | --- | --- | --- |
| Age | -0.10 | 0.01 | -7.52 | <0.01 | -0.13 | -0.07 |
| Obs. | 260 |  |  |  |  |  |
| Mc Fadden’s R^2^ | 0.26 |  |  |  |  |  |
| Mc Fadden’s Adjusted R^2^ | 0.25 |  |  |  |  |  |

N= 260.
