# Supplementary material for: The effect of poor vision on economic farm performance: Evidence from rural Cambodia
Source: PLoS One. 2022 Sep 9;17(9):e0274048. doi: 10.1371/journal.pone.0274048 (PMC9462746; doi:10.1371/journal.pone.0274048)
Supplement: Attachment — Submitted filename: response to reviewer 2.docx [file pone.0274048.s004.docx]

Point-to-point response to **reviewer #2**

Regarding the original research article

**“The effect of poor vision on economic farm performance: Evidence from rural Cambodia.”**

Dear reviewer #2,

Thank you very much for your helpful feedback. We are very grateful for the time and effort that you have devoted to our paper, and we are confident that our revisions and responses below address your concerns. However, we stand ready to make any additional changes that you deem necessary. Please find below our detailed responses (in italics) to your comments (repeated in plain typeface).

**Reviewer #2:**

The paper focuses on a topic with rather unique approach. The correlation of vision problems and farm management is a less researched field so no doubt, the paper is original. Regarding the context, it is obvious that Cambodia is just a kind of case study which allows us to estimate the seriousness of the problem in other developing countries with low level of healthcare.

*Thank you very much for this comment, we are very glad to receive this positive comment.*

The statistical analysis uses the appropriate and well selected toolset and completed well, however, despite most elements of the sampling, dataset and statistical tools are described, the general introduction of the methodology is missing. Which methods were used, based on what assumptions and who else used it for similar purposes? These questions should be answered at the beginning of the methodological parts.

*Thank you very much for this comment. We added a paragraph to the methods section, were we introduce the methodology and in which contexts it is used. We also refer to several studies that evaluate impacts on agricultural production which use these methods. On page 13, we added following sentences to the first paragraph:*

*An important problem of causal inference is how to estimate treatment effects in observational studies, where (like an experiment) a group of units is exposed to a well-defined treatment, but (unlike an experiment) no systematic methods of experimental design are used to maintain a control group(46). To circumvent this problem, we apply Mahalanobis distance matching (MDM) and propensity score matching (PSM). Matching involves pairing treatment and comparison units that are similar in terms of their observable characteristics. These matching methods have become popular in impact evaluations and are used in a variety of fields, including to assess impacts related to agricultural production (47–51).*

The introductory chapter highlights the reason and context of the research well, and also includes some literature review. However, this latter is far not enough. I recommend to write a separate literature review chapter where the related sources are processed analytically, critically and in a comparative way.

*Thank you very much for this comment. As you suggested, we included “Section 2. Literature review” to the manuscript. In this literature review, we discuss 15 studies on the global prevalence of vision impairments and their impacts on global productivity. We critically review the research gaps, with a special attention on the impacts of visual impairments on agricultural productivity. For reasons of brevity we don’t include the chapter here, but kindly refer to the literature review beginning on page 4 of the manuscript.*
